# Disrupted Skies: How Offshore Wind Farms Alter Flight Behavior of Breeding Seabirds

**DOI:** 10.1002/ece3.74089

**Published:** 2026-07-29

**Authors:** Waner Liang, Yanyan Li, Yifei Jia, Shengwu Jiao, Li Wen, Guangchun Lei, Kexun Lou, Xinyi He, Jingwen Cui

**Affiliations:** ^1^ School of Ecology and Nature Conservation Beijing Forestry University Beijing China; ^2^ Center for East Asian‐Australasian Flyway Studies Beijing Forestry University Beijing China; ^3^ Research Institute of Subtropical Forestry Chinese Academy of Forestry/Wetland Ecosystem Research Station of Hangzhou Bay Hangzhou Zhejiang China; ^4^ NSW Department of Planning, Industry and Environment Science, Economics and Insights Division Sydney New South Wales Australia; ^5^ Fuyang District Agricultural Technology Extension Center Hangzhou Zhejiang China; ^6^ College of Forestry Beijing Forestry University Beijing China

**Keywords:** avoidance behavior, breeding conservation, high‐resolution tracking data, offshore wind farms, *Onychoprion anaethetus*, renewable energy

## Abstract

Offshore wind farms are expanding rapidly as part of global climate mitigation efforts, but their effects on seabird movement behavior remain incompletely understood. While collision risk has received substantial attention, less is known about how turbines may alter flight routes through evasive behavior and meso‐avoidance, particularly near breeding colonies where repeated commuting flights may accumulate energetic costs. We investigated flight responses of breeding Bridled Terns ( *Onychoprion anaethetus* ) to offshore wind turbines near their colony using high‐resolution satellite tracking data collected at 1‐s intervals and lower‐resolution data collected at 1‐h intervals. We quantified within‐trajectory flight traits, including mean redirection, number of turns, flight speed, and flight altitude, in relation to turbine exposure. We assessed avoidance using both proximity‐based and direction‐sensitive metrics. At the near‐colony scale, we tested whether flight behavior changed with increasing alignment between the trajectory bearing and turbine bearing from the colony. At the broader breeding‐range scale, we tested whether behavior differed inside and outside wind farms or with distance to turbines, while accounting for colony distance, wind, and landscape variables. Bridled Terns showed increased mean redirection and lower flight altitude when trajectories were more closely aligned with turbine directions from the colony, suggesting localized route alteration in obstacle‐facing directions. However, flight behavior was not significantly associated with turbine proximity, nor did it differ significantly inside and outside wind farms. These findings suggest that offshore wind farms may influence seabird movement through localized, direction‐dependent route alteration rather than simple distance‐dependent responses, highlighting the value of movement‐context metrics and within‐trajectory traits in wind farm impact assessments.

## Graphical abstract

Using high‐resolution 1‐s tracking bursts collected every hour, we quantified within‐trajectory flight behavior of breeding Bridled Terns near offshore wind farms. Birds showed increased mean redirection and lower flight altitude when trajectories were more closely aligned with turbine directions from the colony, suggesting localized, direction‐dependent route alteration. In contrast, flight behavior was not significantly explained by turbine proximity or by whether trajectories occurred inside or outside wind farms.

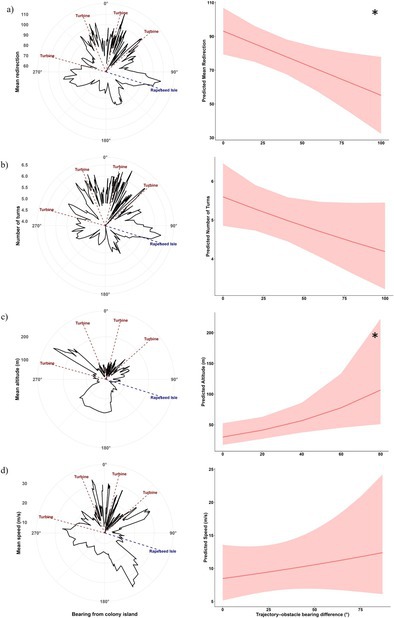

## 1 Introduction

The expansion of wind farms has raised increasing concern about their consequent impact on biodiversity (Galparsoro et al. 2022; Huso et al. 2021). Since the Paris Agreement of the Climate Change Convention (Paris Agreement 2015) and the United Nations' global sustainable development goals (United Nations 2024) have been set, there have been worldwide endeavors to expand the utilization of renewable energies. Among those endeavors, the construction of wind farms is considered one of the options with the most potential (Long et al. 2023; Owusu and Asumadu‐Sarkodie 2016; Zhang et al. 2017). Coastal and offshore regions, with their favorable wind conditions, are particularly attractive for development. However, these areas often coincide with key habitats for migratory waterbirds, creating spatial overlap that can lead to displacement, collision risk, and behavioral disruption (Hoover and Morrison 2005; Kamata et al. 2023; Maurer 2016; Langston and Pullan 2003; Therkildsen et al. 2021; Tolvanen et al. 2023). Important as it is to mitigate climate change and reduce emissions (Bellard et al. 2012; Hoffmann and Sgrò 2011; Parmesan and Yohe 2003), it is equally important to conserve biodiversity. Although renewable energy development is necessary to reduce long‐term climate‐driven biodiversity loss, its local ecological impacts must also be assessed so that climate mitigation does not create avoidable conservation costs.

Many birds respond to wind farms by avoidance (Fielding et al. 2022; Tolvanen et al. 2023; van Bemmelen et al. 2024). According to the framework proposed by May (2015), avoidance behaviors to wind turbines can be categorized into macro‐, meso‐, and micro‐avoidance. Macro‐avoidance describes the long‐term effect on the avoidance of the entire wind farm, often in the form of displacement; meso‐avoidance describes anticipatory evasion (choosing between flight routes) and impulsive evasions (changes within a flight trajectory, e.g., directional change in a trajectory) of a single wind turbine or an array of wind turbines; Micro‐avoidance describes the escape from rotating blades (May 2015; Thaxter et al. 2018). Most studies focused on macro‐avoidance due to its high availability of data (Marques et al. 2020; Mendel et al. 2019; Thaxter et al. 2015; Vanermen et al. 2013; Welcker and Nehls 2016). Meso‐ and micro‐avoidance require higher resolution data due to the short time interval and small spatial scale of those behaviors.

Current studies on meso‐avoidance often focus on displacement at a finer scale (Pollock et al. 2024; Skov et al. 2026), that is, meso‐avoidance as reduced use near turbines. The within‐trajectory evasion behavior aspect of meso‐avoidance, which is characterized by abrupt changes within flight trajectories (May 2015), is still rarely studied. The advance of technology in tracking devices has made high‐resolution tracking data increasingly obtainable. However, to study such evasion behavior of an individual wind turbine, or of an array of wind turbines, the resolution of the data might need to be especially high since the evasion behavior might happen within seconds. Although 1‐s interval tracking data can be collected continuously when animals can carry larger tracking devices with higher battery capacities, and when attachment methods and local weather conditions provide sufficient charging efficiency, battery limitations generally prevent continuous collection of 1‐s interval or higher‐frequency data (Johnston et al. 2022; Thaxter et al. 2018). In the current literature using high‐resolution tracking data, rarely are studies' data as fine as 1‐s interval (Marques et al. 2020; Mendel et al. 2019; Pollock et al. 2024; Thaxter et al. 2015; Vanermen et al. 2013; Welcker and Nehls 2016). Here, we moved away from conventional fixed‐interval tracking and instead used a two‐scale sampling design. By collecting 1‐s interval data for 10 s every hour, we captured fine‐scale flight behavior while preserving battery life. These short high‐frequency bursts provided one trajectory per hour at a resolution sufficient to analyze within‐trajectory behavioral changes and identify potential evasive responses.

We introduce two trajectory traits—mean redirection and number of turns—to quantify tortuosity of the trajectories on a Bridled Tern ( *Onychoprion anaethetus* ) colony on Youbeng Isle, China, where offshore wind turbines have been constructed in close proximity to the breeding colony (~800 m). Along with altitude and speed, we aim to use those trajectory traits to examine the behaviors of Bridled Terns: (1) to the obstacles near their breeding colony, (2) to individual turbines within their breeding range, and (3) within and outside wind farms.

## 2 Materials and Methods

### 2.1 Study Area

**FIGURE 1:**
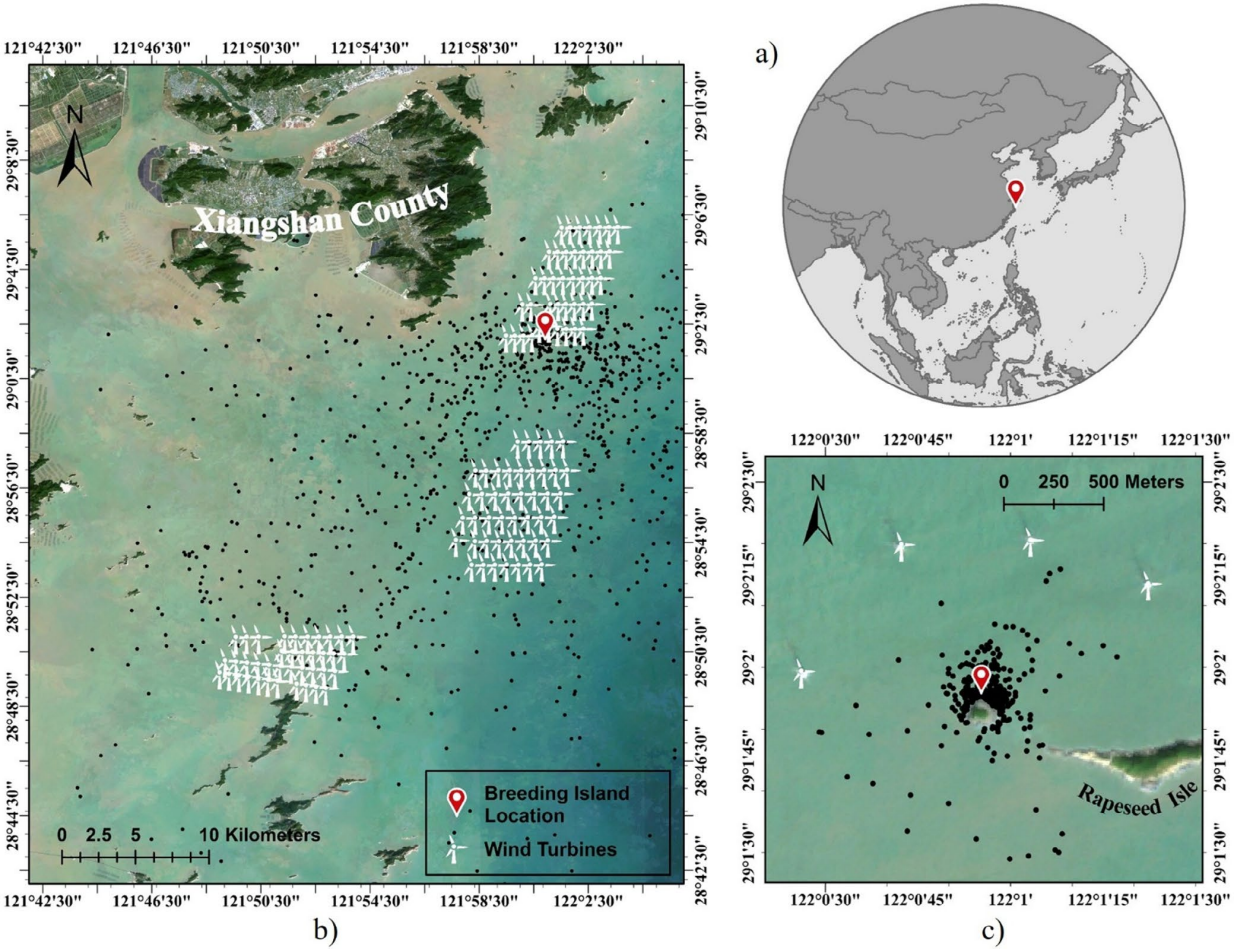
Study area. (a) Location of the study area. (b) The entire breeding range, excluding foraging grounds. Black points indicate trajectories consisting of 10 location fixes collected at 1‐s intervals, with one trajectory recorded every hour. (c) The near‐colony area, with Rapeseed Isle marked. Black points indicate trajectories consisting of 10 location fixes collected at 1‐s intervals, with one trajectory recorded every hour. Year‐mean satellite image from 2023 obtained from Sentinel‐2 were used as the base maps in b and c.

The study focuses on a breeding colony of Bridled Terns on Youbeng Isle (油甏屿, or YouBeng Yu, 122.0151591° E, 29.0313404° N), Xiangshan county, Zhejiang Province, China (Figure 1a). Youbeng Isle lies approximately 6 km from the nearest shore. It mainly supports two seabird species, the Bridled Tern and the Black‐tailed Gull ( *Larus crassirostris* ). There are over 1000 breeding pairs of Bridled Terns on Youbeng Isle each year. The isle is in a protected area named the Specially Protected Islands East of Nantian (南田东侧特别保护海岛), where exploitation of resources and wildlife is prohibited.

In the colony's southeast vicinity lies a larger island, Rapeseed Isle (油菜花屿, or Youcaihua Yu, 122.0229900° E, 29.0290127° N), where the boundary of an offshore wind farm surrounds its west, north to northeast direction, leaving only the southwest direction clear of obstacles (Figure 1b,c). The wind farm, Xiangshan No. 1 Offshore Wind Farm (Phase I), that was developed by China Guodian, was completed and put to use on December 24, 2021, with a length of north and south of 6.3 km, east and west of the widest 5.9 km, a planning area of about 28 km^2^, an assembly of 41 sets of 6.2 MW fan wind turbines, and a total installed capacity of 254.2 MW (Ningbo Municipal Government 2024). The spacings between the wind turbines are about 450–800 m, and the spacings between rows are about 1.6–2 km. The nearest wind turbine is less than 800 m from the breeding colony. The hub height of the wind turbine was either 109.9 or 104.5 m, with a rotor radius of 85.5 m and estimated rotor swept zones of 19.0–195.4 m.

Two more wind farms were within the breeding range of the Bridled Tern colony, with approximately 7 and 23 km away from the colony, respectively (Figure 1b). The locations of wind turbines were manually digitized from high‐resolution satellite imagery. Turbine positions were identified visually based on turbine structures visible in the imagery. The same turbine‐location dataset was used consistently for figure preparation and all spatial analyses.

In this study, we focused on three spatial scales: (a) the near‐colony area within 800 m of the center of Youbeng Isle (Figure 1c); (b) areas within 800 m of turbines across the breeding range; and (c) the broader breeding range, including the three wind farms but excluding foraging grounds (Figure 1b; 28.7°–29.2° N, 121.58°–122.10° E). The foraging ground was identified through water depth. Bridled Terns are known to make long foraging trips up to 20–80 km over mid and outer shelf waters during their breeding season (Dunlop 1997; Dunlop and Surman 2012), and shallow water is not their typical foraging habitat. Based on bathymetric data acquired from Sentinel‐2 Level‐2A, surface reflectance imagery was used to estimate bathymetry within the study area. Our entire study area in Figure 1b has a water depth below 20 m, and we estimate the Bridled Tern colony's main foraging range would be at least 30 km to the east of the colony. By excluding the foraging ground, we expect that foraging behavior had minimal effect on the tortuosity of flight trajectories within our study area.

### 2.2 Bird Tagging

Bridled Tern is a marine species of the Onychoprion genus with a worldwide distribution in warm to tropical waters and a large population of 400,000 to 1,000,000 (BirdLife 2019; Delany et al. 2006). This species breeds on continental islands instead of oceanic islands (Dunlop 1997), which makes it exposed to offshore wind farm construction.

In 2023, three adult Bridled Terns were captured and tagged at Youbeng Isle. Satellite tags from Hunan Global Messenger Technology Co. Ltd. (model HQBG1204, weighing 4.8 g) were attached from their mantle to rump. Data from July to September 2023 were used in this study.

**FIGURE 2:**
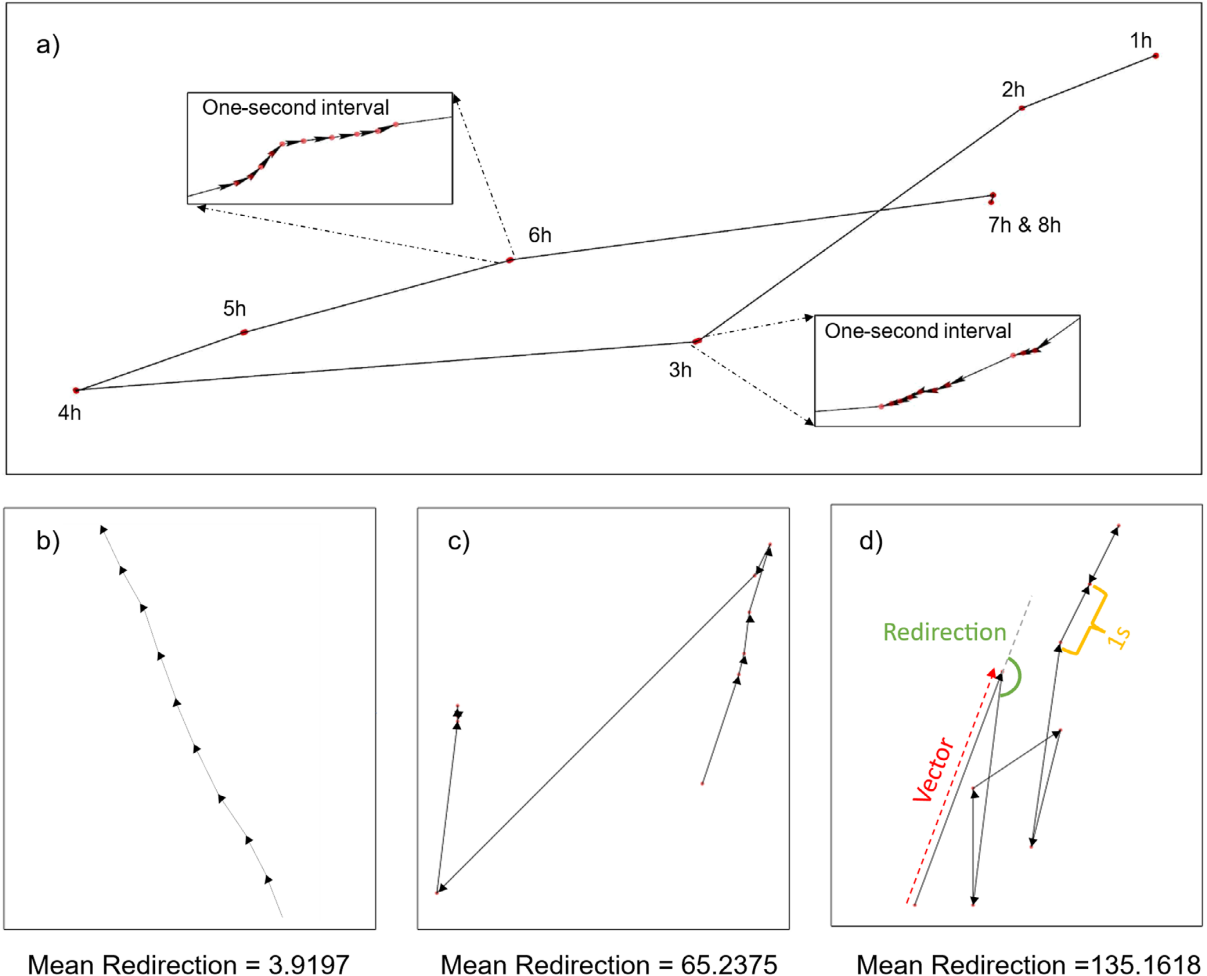
Diagrams explaining the mean redirection. (a) Example 8‐h trajectories recorded at 10‐s intervals, with inset panels highlighting the third and sixth trajectories. (b–d) Example trajectories representing three characteristic levels of mean redirection. At values near 0°, trajectories show clear directionality with few sharp turns; at values near 65°, sharp turns are more evident, but overall direction remains discernible; at values near 135°, trajectories appear more tortuous, and directional movement is less clear. Terms and time intervals are illustrated in panel d.

The satellite data were recorded at two temporal scales, where time intervals were 1 h and 1 s, respectively. Ten location fixes of 1‐s intervals were recorded by the tracker every hour. We call this 10‐s data sequence a “trajectory,” whose high‐throughput data will later be used for behavioral analysis (Figure 2a). This allows for more detailed behavioral analysis than obtaining a single location fix per hour. Only high‐accuracy satellite data, that is, coordinates obtained using a minimum of seven satellites and a residual error value of less than 10 m (Fleming et al. 2020), were used for further analysis of altitude and speed.

### 2.3 Data Preparation

#### 2.3.1 Directional Changes

A trajectory could range from being consistently straight, with each step facing a similar direction, to having acute angles and sharp turns. These features can be described using quantified directional changes in a trajectory, that is, whether or not the individual made a “notable” change in its flying direction. Directional changes can be used to assess the Bridled Tern's behavior toward different obstacles surrounding Youbeng Isle. To quantitatively describe what kind of directional change is “notable,” we defined two trajectory traits, namely mean redirection and number of turns.

Mean redirection was defined as the mean change of direction in a trajectory. Each trajectory contains 10 consecutive locations with 1‐s intervals. We calculated vectors for consecutive locations, resulting in nine vectors. We then calculated the difference in direction between each consecutive vector as Redirections. Between two vectors, the difference of the directions can either be calculated clockwise or counterclockwise; we use the absolute value of the smaller difference as the redirection. Therefore, the mean redirection is:

(1) $$\text{Mean Redirection}=\frac{\sum_{i=1}^{8}{\text{Redirection}}_{i}}{8}$$

where redirection is the *i*th direction change. Out of 10 location points, there would be nine vectors and eight direction changes.

The mean redirection, in our case, ranged roughly from 0° to 178°, where zero degrees would mean no change in direction at all (i.e., the tagged individual stayed in a straight line during the entire trajectory), and larger degrees would mean tortuous movement (Figure 2).

The number of turns was defined as the number of redirections that were larger than 30°. Thirty degrees was an empirical value, where when a turn was larger than 30°, we considered the tagged individual had made a notable turn in its flight direction.

Mean redirection and number of turns show different aspects of a trajectory: mean redirection describes the average directional change, whereas number of turns describes the frequency of turns.

#### 2.3.2 Locations and Bearings of Trajectories

We calculated the geometric center of a trajectory to represent its spatial location. The bearing of trajectories was calculated as the bearing from the geometric center of the island to the trajectories.

#### 2.3.3 Wind Interference

Hourly crosswind and tailwind components were calculated for each fix based on its time and location and then averaged for each trajectory to account for wind conditions in our analysis. We downloaded the eastward and northward components (U‐component and V‐component, respectively) and the wind speed of the 10‐m wind from the “ERA5‐Land Hourly ‐ ECMWF Climate Reanalysis” dataset (Muñoz Sabater 2019) using Google Earth Engine. The U‐component and V‐component were used to calculate the wind direction. Wind direction was calculated using Equation (2), where *u* is the U‐component and *v* is the V‐component, WD is wind direction:
(2)

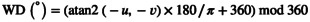




Tailwind and crosswind components were then calculated by comparing wind direction with the movement direction of each fix. The angular difference between wind direction and movement direction was calculated as follows:

(3) θ=WD−BD

where WD is wind direction, and BD is bird movement direction, both expressed in degrees. The angular difference was converted from degrees to radians before calculating wind components.

Tailwind was calculated as follows:

(4) $$\text{Tailwind}=\mathrm{WS}\times \cos\left(\theta \right)$$

Crosswind was calculated as follows:

(5) $$\text{Crosswind}=\mathrm{WS}\times \sin\left(\theta \right)$$

where WS is wind speed, and *θ* is the angular difference between wind direction and movement direction.

### 2.4 Analysis

#### 2.4.1 Near‐Colony Directional and Proximity Analysis

This analysis examined Bridled Tern behavior in relation to the four wind turbines surrounding the colony and Rapeseed Isle. The nearest wind turbine was approximately 800 m from the center of the colony island. Rapeseed Isle was approximately 400 m from the colony center at its nearest point, and approximately 800 m from the colony center to the center of the island. This analysis included trajectories within 800 m from the center of the colony and excluded trajectories on top of the colony island to exclude the influence of the on‐island terrain, resulting in a total of 422 trajectories (*n* = 241, *n* = 86, *n* = 95 for each individual, respectively).

For each trajectory, the trajectory–obstacle bearing difference was calculated as the angular difference between the bearing from the colony center to the trajectory and the bearing from the colony center to each obstacle, including the four wind turbines and Rapeseed Isle. This measure describes how closely each trajectory aligned with the direction of an obstacle from the colony center.

The smallest trajectory–obstacle bearing difference across the four wind turbines and Rapeseed Isle was used to assess whether trajectories that were more closely aligned with obstacle directions differed in mean redirection, number of turns, altitude, or speed. The minimum distance to an obstacle was included to examine whether these response variables changed with proximity to obstacles. The nearest obstacle, defined as the obstacle with the smallest trajectory–obstacle bearing difference, was included to test whether behavioral responses differed among obstacles.

Standardized tailwind, standardized crosswind, and bird ID were also included as fixed effects to account for wind conditions and individual differences. Tailwind and crosswind were standardized by centering each variable on its mean and scaling by its standard deviation. Bird ID was included as a fixed effect rather than a random effect because only three individuals were available for analysis.

Mean redirection, number of turns, altitude, and speed were modeled as separate response variables. Mean redirection and altitude were modeled using linear models:

$$\begin{array}{c} \text{response}\sim \text{trajectory}–\text{obstacle bearing difference}\\ +\text{minimum distance to obstacle}+\text{nearest obstacle}\;\mathrm{ID}\\ +\text{standardized tailwind}+\text{standardized crosswind}+\text{bird}\;\mathrm{ID} \end{array}$$

Speed was modeled using a generalized additive model, with the same fixed effects. The number of turns was modeled using a zero‐inflated COM‐Poisson model, also using the same fixed effects.

#### 2.4.2 Near‐Turbine Proximity Analysis

To examine whether flight behavior varied with proximity to turbines across the broader breeding range, we conducted a secondary analysis using only trajectory segments located within 800 m of the nearest turbine while excluding the trajectories within 200 m of the colony center to reduce the colony‐edge confounding, resulting in 210 trajectories (*n* = 97, *n* = 49, *n* = 64, respectively for the three individuals). This subset was used to focus the analysis on flights occurring in the vicinity of turbines, rather than including distant trajectories for which turbine proximity was unlikely to represent a meaningful exposure variable. Unlike the colony‐centered directional analysis, which focused on turbines located near the breeding colony, this analysis assessed proximity‐related responses across all turbines in the breeding range.

Minimum distance to the turbine was used as the main predictor. Distance to colony, standardized tailwind, standardized crosswind, and bird ID were included as fixed effects to account for colony proximity, wind conditions, and individual differences.

Mean redirection, number of turns, altitude, and speed were modeled as separate response variables. Altitude was modeled using linear models:

$$\begin{array}{c} \text{response}\sim \text{minimum distance to turbine}\\ +\text{distance to colony}+\text{standardized tailwind}\\ +\text{standardized crosswind}+\text{bird}\;\mathrm{ID} \end{array}$$

Mean redirection and speed were modeled using a generalized additive model, with the same fixed effects. The number of turns was modeled using a zero‐inflated COM‐Poisson model, also using the same fixed effects.

#### 2.4.3 Behavioral Comparison Within and Outside the Wind Farm

This analysis was conducted on the entire breeding range, excluding the foraging ground. Three wind farms were present in this area. The boundary of each wind farm was defined as the contour formed by the outermost turbines. Trajectories were then classified according to whether they occurred inside or outside a wind farm, resulting in 209 trajectories inside the wind farms and 993 trajectories outside the wind farms.

Trajectory location, defined as inside or outside a wind farm, was used as the main predictor. Standardized tailwind, standardized crosswind, and bird ID were included as fixed effects to account for wind conditions and individual differences.

Mean redirection, number of turns, altitude, and speed were modeled as separate response variables. Altitude and speed were modeled using linear models:

$$\begin{array}{c} \text{response}\sim \text{location}+\text{standardized tailwind}\\ +\text{standardized crosswind}+\text{bird}\;\mathrm{ID} \end{array}$$

Mean redirection was modeled using a generalized additive model, with the same fixed effects. The number of turns was modeled using a zero‐inflated COM‐Poisson model, also using the same fixed effects.

R packages zoo (Zeileis and Grothendieck 2005), lubridate (Garrett and Hadley 2011), geosphere (Hijmans 2024), sf (Pebesma 2018; Pebesma and Bivand 2023), dplyr (Wickham et al. 2023), mgcv (Wood 2017), glmmTMB (Mollie et al. 2017), emmeans (Lenth and Piaskowski 2026), DHARMa (Hartig 2024), ggeffects (Lüdecke 2018), and ggplot2 (Wickham 2016) were used in the analysis and visualization. Analyses were conducted on R version 4.5.1.

## 3 Results

### 3.1 Near‐Colony Behavior Responses to Obstacles

**FIGURE 3:**
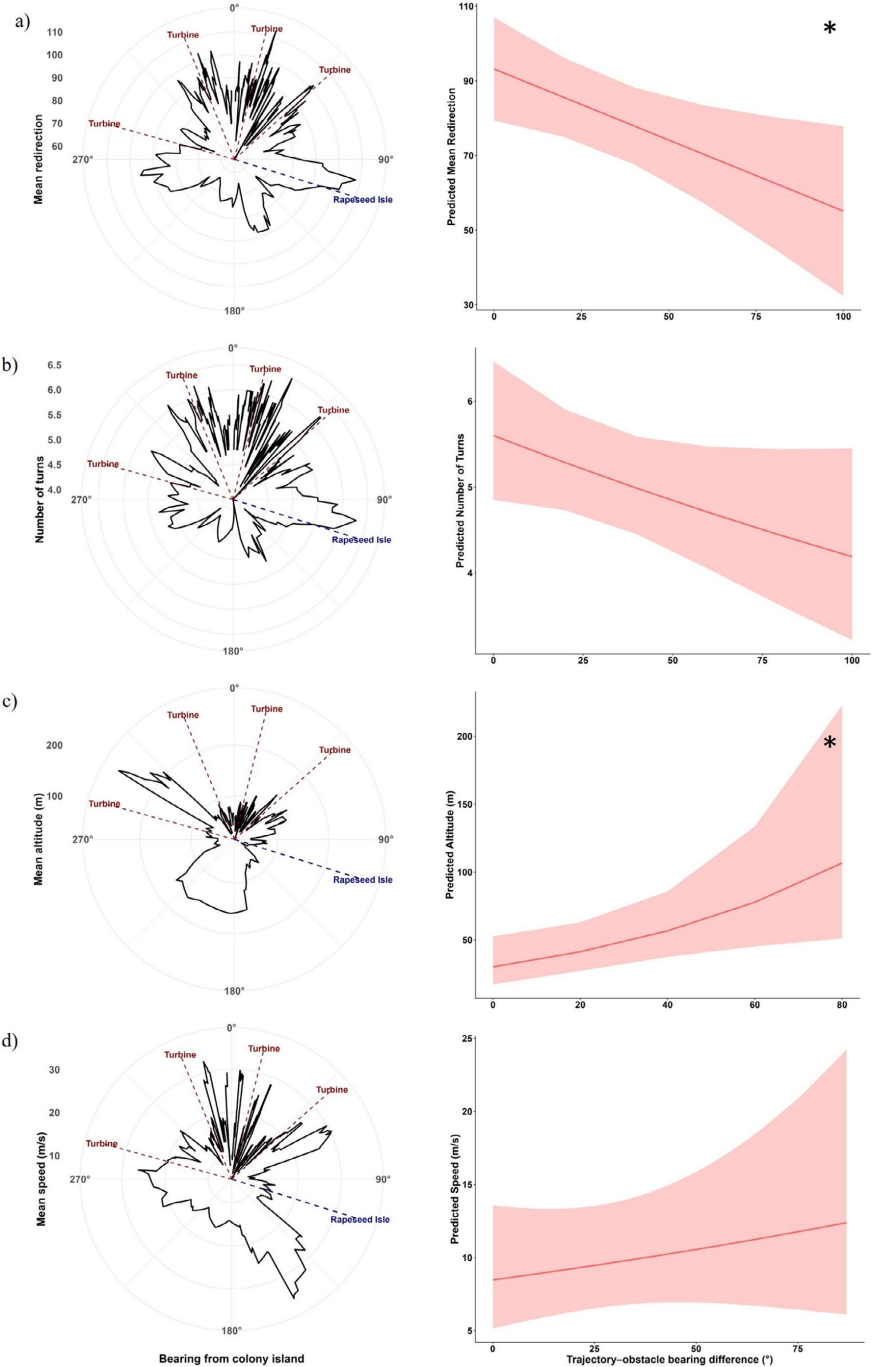
Relationship between trajectory–obstacle bearing difference and flight behavior. Asterisks indicate statistical significance at *p* < 0.05. Left panels show rolling means of 10‐degree angular intervals, with obstacle bearings marked with dashed lines. Right panels show model‐predicted values of each behavioral response in relation to trajectory–obstacle bearing difference, with other covariates held constant. The line shows model‐predicted response variables, and the shaded area shows the 95% confidence interval.

Trajectory–obstacle bearing difference was significantly associated with mean redirection and altitude (Figure 3). As trajectories became more closely aligned with obstacles, mean redirection increased, and altitude decreased. For each 1° decrease in trajectory–obstacle bearing difference, mean redirection increased by 0.38° and altitude decreased by 0.41 m.

There was no evidence that the number of turns or speed changed with trajectory–obstacle bearing difference. Although the predicted number of turns declined as trajectories became more closely aligned with obstacles, this relationship was not statistically significant. Therefore, directional responses to obstacles were supported for mean redirection and altitude, but not for discrete turn frequency or speed.

There was no evidence that minimum distance to obstacle affected any response variable (Table S1).

There was no evidence that behavioral responses differed significantly among obstacles for any response variable (Table S2). This indicates that Bridled Terns did not show detectable differences in response among individual wind turbines or between turbines and Rapeseed Isle.

**FIGURE 4:**
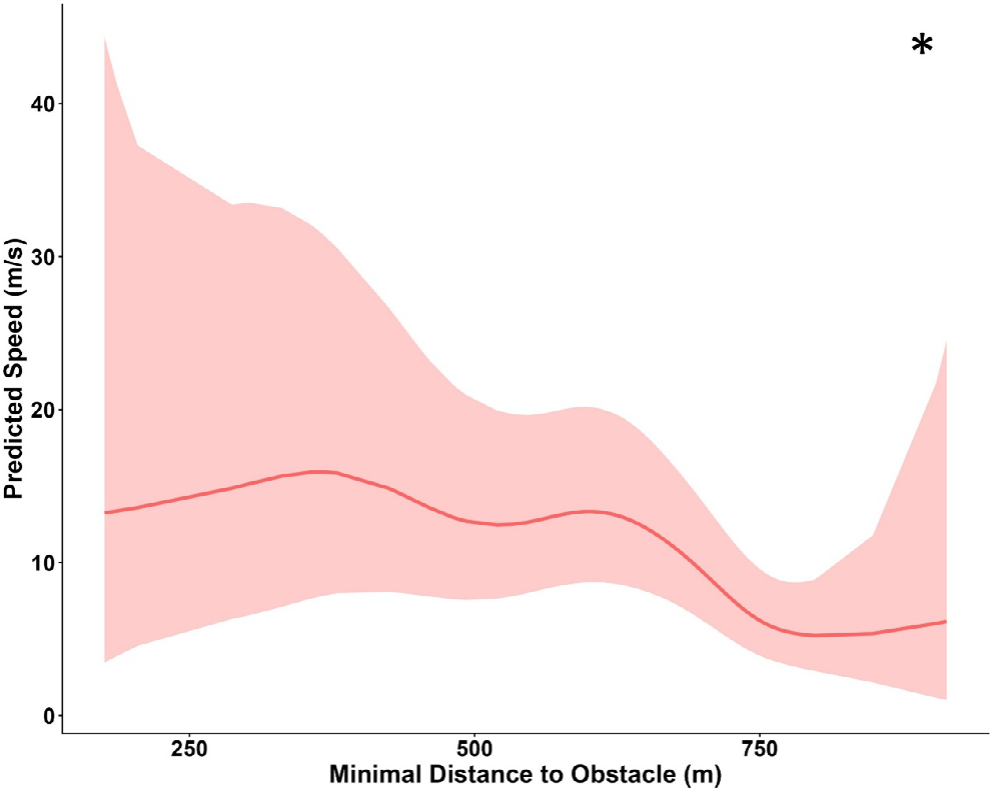
Predicted non‐linear relationship between minimum distance to the obstacle and flight speed. Asterisks indicate statistical significance at *p* < 0.05. Other covariates were held constant. The line shows model‐predicted speed, and the shaded area shows the 95% confidence interval.

Minimum distance to the nearest obstacle was not significantly associated with mean redirection, number of turns, or altitude, but showed a significant non‐linear relationship with speed (Figure 4).

Overall, Bridled Terns flying in obstacle‐facing directions showed higher mean redirection and lower flight altitude, with no clear evidence of corresponding changes in speed or number of turns.

### 3.2 Near‐Turbine Behavioral Analysis

For trajectories within 800 m of turbines across the breeding range, there was no evidence that mean redirection, number of turns, altitude, or speed varied with distance to the nearest turbine (Table S3).

### 3.3 Behavioral Comparison Within and Outside the Wind Farm

There was no evidence that mean redirection, number of turns, altitude, or speed differed between trajectories inside and outside wind farms (Table S4).

## 4 Discussion

This study provides empirical evidence that offshore wind farms can be associated with altered flight behavior in breeding Bridled Terns through meso‐scale route alteration. By using high‐resolution 10‐s trajectory bursts, we quantified within‐trajectory directional changes rather than relying solely on point‐based proximity or wind farm occupancy metrics. We examined trajectory‐level behavioral responses in relation to both obstacle proximity and colony‐centered directional alignment with turbines and natural barriers. This approach provides insight into how seabirds interact with wind energy infrastructure at operational sites, particularly where turbines are located close to breeding colonies and regular commuting routes.

### 4.1 Speed and Mean Redirection

Flight speed showed a significant non‐linear relationship with proximity to obstacles, but not with alignment to obstacles. However, the apparent dip in speed occurred approximately 750 m from obstacles, which coincided with the distance to the breeding colony. This suggests that the speed pattern may reflect colony‐associated flight behavior rather than a direct response to obstacles.

In addition, speed was not strongly correlated with mean redirection across trajectories within the breeding range (*r* = 0.09), suggesting that increased trajectory tortuosity was not simply associated with changes in flight speed.

### 4.2 Meso‐Avoidance to Near‐Colony Obstacles

The increase in mean redirection and decrease in flight altitude with increasing alignment between trajectory bearing and turbine bearing from the breeding colony suggest that Bridled Terns altered their flight behavior when moving in turbine‐facing directions. This pattern is consistent with meso‐scale avoidance or evasive flight behavior reported in previous radar‐ and tracking‐based studies (Fielding et al. 2022; Mendel et al. 2019). Increased redirection may indicate greater route adjustment around turbines, although the underlying behavioral mechanism cannot be determined directly from trajectory geometry alone. The decrease in flight altitude in turbine‐facing directions may potentially alter birds' vertical overlap with the rotor‐swept zone of 19.0–195.4 m, although this remains difficult to interpret due to the lack of evidence on proximity in both the near‐colony and near‐turbine analyses.

**FIGURE 5:**
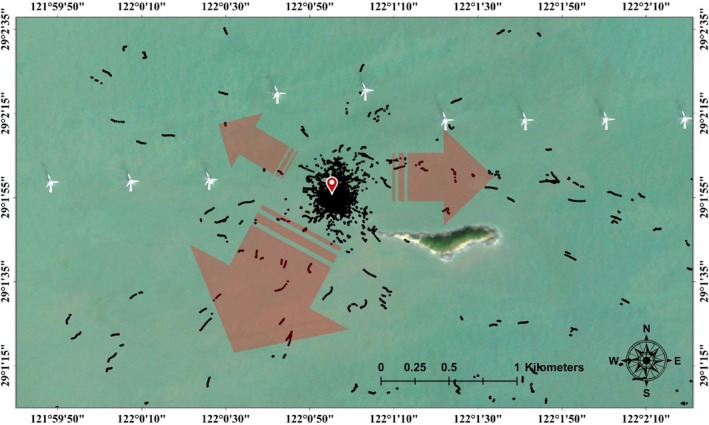
Potential flight routes out of the breeding colony. Black points represent raw 10‐s fixes sampled hourly (not trajectories). Red arrows represent potential main flight routes where the angular difference between obstacles was large.

The increase in flight tortuosity with greater turbine alignment further suggests that birds did not respond to the turbine arrays as a single continuous barrier. Instead, their flight trajectories appeared to be modified in relation to turbine‐facing directions, consistent with localized route alteration around individual turbines or turbine clusters. We found no evidence that avoidance responses differed significantly among turbines or between turbines and Rapeseed Isle, suggesting that individual turbines may have acted as local movement obstacles with barrier effects comparable in magnitude to those associated with the nearby island (Figure 5).

Although the energetic consequences of these behavioral changes were not measured directly, repeated evasive movements and detours during flights to and from the breeding colony could accumulate over the breeding season. Such cumulative route alteration may increase travel costs for seabirds, particularly when wind turbines are located close to breeding colonies and regular commuting routes. These findings suggest that turbine layout geometry, including spacing and angular width relative to colony‐based flight directions, may play an important role in shaping flight paths and potential energetic costs during foraging trips.

Because the analysis was based on a limited number of tracked individuals, these results should be interpreted as evidence of behavioral responses in this study system rather than as a population‐level estimate of wind farm effects.

### 4.3 Direction‐Sensitive Measures of Meso‐Avoidance Might Complement Conventional Proximity‐Based Measures

Proximity and angle of approach have been identified as important predictors of meso‐avoidance responses to wind turbines (May 2015). In this study, we considered both spatial proximity and directional context, although our directional metric was not a direct measure of instantaneous approach angle. Instead, we examined the angular difference between trajectory bearing and obstacle bearing from the breeding colony, providing a colony‐centered measure of whether birds moved in turbine‐facing directions.

Proximity‐based analyses showed no significant association between flight behavior and distance to turbines at either the near‐colony scale or within 800 m of the nearest turbine. We also found no significant trajectory‐level behavioral differences between flights inside and outside wind farms. Together, these results suggest that movement context may provide information not captured by conventional proximity‐based exposure metrics. In particular, how a bird is moving in relation to turbines may be more informative than where it is located relative to turbines alone.

Future studies could build on this approach by classifying sub‐trajectories according to turbine‐relative movement direction, such as flying toward, away from, or past turbines, to better distinguish proximity from approach context and improve detection of meso‐avoidance behavior.

However, the absence of significant proximity‐based effects should not be interpreted as evidence that turbines had no influence on movement, or as evidence of a displacing effect, but rather that behavioral variation was not well explained by simple distance‐based exposure metrics.

### 4.4 Wind Farm Planning

For wind farm planning, these results suggest that assessments should consider colony location, regular flight corridors, and turbine layout geometry, rather than relying only on turbine buffers or wind farm boundaries. According to our results on angular alignment with obstacles, increasing the size of the gaps of turbines might create a passage that reduces meso‐avoidance intensity. This selective use of open flight paths between wind turbines has been observed in our data (Figure 5) and also in other seabird species (Christensen et al. 2004; Johnston et al. 2022). However, this should be treated with caution, as this might invite Bridled Terns into the wind farms and create ecological traps—situations where increased bird activity within wind farms heightens collision risk (May 2015; Russo et al. 2024; Wang et al. 2015). Adaptive design thus requires optimizing spacing to support coexistence without increasing hazard exposure.

The observed behavioral responses underscore the need to integrate movement ecology into offshore wind development policy. As global wind capacity expands, spatial overlap between infrastructure and seabird habitats will intensify (Galparsoro et al. 2022; IEA 2024). Proactive spatial planning, informed by empirical tracking data and species‐specific sensitivity assessments, is vital to preventing cumulative ecological degradation. Designing energy systems that maintain both ecological integrity and societal trust is central to achieving sustainable marine coexistence.

## Author Contributions

**Waner Liang:** conceptualization (lead), data curation (lead), formal analysis (lead), methodology (lead), visualization (lead), writing – original draft (lead), writing – review and editing (lead). **Yanyan Li:** data curation (lead), methodology (lead), visualization (lead), writing – original draft (lead), writing – review and editing (lead). **Yifei Jia:** conceptualization (supporting), data curation (supporting), funding acquisition (lead), investigation (supporting), methodology (equal), project administration (lead), resources (lead), supervision (lead), visualization (supporting), writing – original draft (supporting), writing – review and editing (supporting). **Shengwu Jiao:** data curation (equal), investigation (equal), writing – original draft (equal), writing – review and editing (equal). **Li Wen:** conceptualization (supporting), data curation (supporting), formal analysis (supporting), methodology (equal), software (supporting), visualization (supporting), writing – original draft (supporting), writing – review and editing (supporting). **Guangchun Lei:** writing – review and editing (supporting). **Kexun Lou:** data curation (supporting), investigation (equal). **Xinyi He:** investigation (supporting). **Jingwen Cui:** investigation (equal), resources (supporting).

## Funding

This work was supported by the National Key Research and Development Program of China (grant number 2022YFF1303402); National Natural Science Foundation of China (grant number 4236114487); and Beijing Forestry University “National Training Program of Innovation and Entrepreneurship for Undergraduates” (grant number S202410022210).

## Ethics Statement

The capturing and tagging procedures used in this study were conducted under *Ci Lin Hu Permit 2021 No. (01)*. All procedures were conducted in accordance with regulations and guidelines.

## Conflicts of Interest

The authors declare no conflicts of interest.

## Supporting information


**Table S1:** Effect of minimum distance to nearest obstacle in the near‐colony analysis.
**Table S2:** Tukey‐adjusted pairwise comparisons among nearest obstacles.
**Table S3:** Effect of minimum distance to nearest turbine within 800 m of turbines.
**Table S4:** Effect of inside/outside wind farm classification on flight behavior.

**Data S1:** Location of the wind turbines.

**Data S2:** Main data.

**Data S3:** README.

## Data Availability

The data supporting the findings of this study are available in the Supporting Informations and are provided as CSV files.
