# Supplementary material for: Disrupted Skies: How Offshore Wind Farms Alter Flight Behavior of Breeding Seabirds
Source: Ecol Evol. 2026 Jul 29;16(8):e74089. doi: 10.1002/ece3.74089 (PMC13418616; doi:10.1002/ece3.74089)
Supplement: Supplementary file 1 — Table S1: Effect of minimum distance to nearest obstacle in the near‐colony analysis. Table S2: Tukey‐adjusted pairwise comparisons among nearest obstacles. Table S3: Effect of minimum distance to nearest turbine within 800 m of turbines. Table S4: Effect of inside/outside wind farm classification on flight behavior. [file ECE3-16-e74089-s003.docx]

**S1. Overview of secondary analyses**

We conducted secondary analyses to test whether trajectory-level flight behaviour was associated with proximity-based exposure metrics or broad wind farm occupancy. These analyses complemented the main near-colony directional analysis and were used to assess whether behavioural responses were explained by simple distance to obstacles, distance to turbines, or inside/outside wind farm classification.

The response variables were mean redirection, number of turns, mean altitude, and mean speed. Tailwind and crosswind components were standardized and included as covariates. Individual identity was included to account for among-individual differences.

**S2. Near-colony proximity and obstacle identity**

For the near-colony analysis, we tested whether behaviour was associated with minimum distance to the nearest obstacle and nearest obstacle identity after accounting for trajectory–obstacle bearing difference, wind conditions, and individual identity.

The model structures were:

Mean redirection:

mean_redirection ~ direction_diff_turbine + distance_nearest_obstacle + closest_object + tailwind_z + crosswind_z + individual_local_identifier

Number of turns:

num_of_turns ~ direction_diff_turbine + distance_nearest_obstacle + closest_object + tailwind_z + crosswind_z + individual_local_identifier

Mean altitude:

log(mean_altitude_m) ~ direction_diff_turbine + distance_nearest_obstacle + closest_object + tailwind_z + crosswind_z + individual_local_identifier

Mean redirection and altitude were analysed using linear models. Number of turns was analysed using a zero-inflated COM-Poisson model.

Minimum distance to the nearest obstacle was not significantly associated with mean redirection, number of turns, or altitude.

Table S1. Effect of minimum distance to nearest obstacle in the near-colony analysis.

| Response variable | Model type | Estimate | SE | Test statistic | P-value |
| --- | --- | --- | --- | --- | --- |
| Mean redirection | Linear model | -0.0009 | 0.0163 | t = -0.052 | 0.958 |
| Number of turns | Zero-inflated COM-Poisson model | 0.00009 | 0.00018 | z = 0.522 | 0.602 |
| Mean altitude | Linear model, log-transformed response | 0.00021 | 0.00066 | t = 0.312 | 0.755 |

Pairwise comparisons among nearest obstacles showed no significant differences among individual turbines or between turbines and Rapeseed Isle.

Table S2. Tukey-adjusted pairwise comparisons among nearest obstacles.

| Response variable | P-value range | Result |
| --- | --- | --- |
| Mean redirection | 0.555–1.000 | No significant pairwise differences |
| Number of turns | 0.477–1.000 | No significant pairwise differences |
| Mean altitude | 0.519–1.000 | No significant pairwise differences |

**S3. Near-turbine proximity analysis**

For trajectories within 800 m of turbines across the breeding range, we tested whether flight behaviour was associated with minimum distance to the nearest turbine after accounting for distance to colony, wind conditions, and individual identity.

The model structures were:

Mean redirection:

mean_redirection ~ s(Min_distance_to_turbine) + s(distance_to_colony) + s(tailwind_z) + s(crosswind_z) + s(individual.local.identifier)

Number of turns:

num_of_turns ~ Min_distance_to_turbine + distance_to_colony + tailwind_z + crosswind_z + individual.local.identifier

Mean altitude:

log(mean_altitude_m) ~ Min_distance_to_turbine + distance_to_colony + tailwind_z + crosswind_z + individual.local.identifier

Mean speed:

log(mean_calculated_speed_m_s + 1) ~ s(Min_distance_to_turbine) + s(distance_to_colony) + s(tailwind_z) + s(crosswind_z) + s(individual.local.identifier)

Mean redirection and speed were analysed using generalized additive models. Altitude was analysed using a linear model. Number of turns was analysed using a zero-inflated COM-Poisson model.

Minimum distance to the nearest turbine was not significantly associated with any response variable.

Table S3. Effect of minimum distance to nearest turbine within 800 m of turbines.

| Response variable | Model type | Effect term | Estimate / edf | SE / Ref. df | Test statistic | P-value |
| --- | --- | --- | --- | --- | --- | --- |
| Mean redirection | GAM | s(Min_distance_to_turbine) | edf = 2.276 | Ref. df = 2.868 | F = 1.142 | 0.292 |
| Number of turns | Zero-inflated COM-Poisson model | Min_distance_to_turbine | 0.00003 | 0.00015 | z = 0.186 | 0.852 |
| Mean altitude | Linear model, log-transformed response | Min_distance_to_turbine | -0.00068 | 0.00065 | t = -1.038 | 0.301 |
| Mean speed | GAM, log(Y + 1)-transformed response | s(Min_distance_to_turbine) | edf = 1.000 | Ref. df = 1.000 | F = 0.250 | 0.618 |

Model diagnostics for the zero-inflated COM-Poisson model of number of turns did not indicate overdispersion or excess zero inflation.

**S4. Inside/outside wind farm comparison**

For the broader breeding-range analysis, trajectories were classified as inside or outside wind farms using polygons defined by the outermost turbines. Likely foraging habitat was excluded from this analysis. The final dataset included 209 trajectories inside wind farms and 993 trajectories outside wind farms.

The model structures were:

Mean redirection:

mean_redirection ~ location + s(tailwind_z) + s(crosswind_z) + s(individual.local.identifier)

Number of turns:

num_of_turns ~ location + tailwind_z + crosswind_z + individual.local.identifier

Mean altitude:

log(mean_altitude_m) ~ location + tailwind_z + crosswind_z + individual.local.identifier

Mean speed:

log(mean_calculated_speed_m_s + 1) ~ location + tailwind_z + crosswind_z + individual.local.identifier

Mean redirection was analysed using a generalized additive model. Altitude and speed were analysed using linear models. Number of turns was analysed using a zero-inflated COM-Poisson model.

There was no evidence that flight behaviour differed between trajectories inside and outside wind farms.

Table S4. Effect of inside/outside wind farm classification on flight behaviour.

| Response variable | Model type | Contrast | Estimate | SE | Test statistic | P-value |
| --- | --- | --- | --- | --- | --- | --- |
| Mean redirection | GAM | Outside vs inside | -1.939 | 2.053 | t = -0.944 | 0.345 |
| Number of turns | Zero-inflated COM-Poisson model | Outside vs inside | -0.0159 | 0.0315 | z = -0.503 | 0.615 |
| Mean altitude | Linear model, log-transformed response | Outside vs inside | -0.0059 | 0.1068 | t = -0.056 | 0.956 |
| Mean speed | Linear model, log(Y + 1)-transformed response | Outside vs inside | -0.0788 | 0.0621 | t = -1.268 | 0.205 |
